# Analysis on Spatio-Temporal Evolution and Influencing Factors of Air Quality Index (AQI) in China

**DOI:** 10.3390/toxics10120712

**Published:** 2022-11-22

**Authors:** Renyi Yang, Changbiao Zhong

**Affiliations:** 1 School of Economics, Yunnan University of Finance and Economics, Kunming 650221, China; 2 Institute of Land & Resources and Sustainable Development, Yunnan University of Finance and Economics, Kunming 650221, China; 3 Institute of Targeted Poverty Alleviation and Development, Yunnan University of Finance and Economics, Kunming 650221, China

**Keywords:** Air Quality Index, spatiotemporal evolution, influencing factors, spillover effect, spatial correlation

## Abstract

After the reform and opening up, China’s economy has developed rapidly. However, environmental problems have gradually emerged, the top of which is air pollution. We have used the following methods: In view of the shortcomings of the current spatio-temporal evolution analysis of the Air Quality Index (AQI) that is not detailed to the county level and the lack of analysis of its underlying causes, this study collects the AQI of all counties in China from 2014 to 2021, and uses spatial autocorrelation and other analysis methods to deeply analyze the spatio-temporal evolution characteristic. Based on the provincial panel data, the spatial econometric model is used to explore its influencing factors and spillover effects. The research results show that: (1) From 2014 to 2021, the AQI of all counties in China showed obvious spatial agglomeration characteristics, and counties in central and western Xinjiang, as well as Beijing, Tianjin, and Hebei, were high-value agglomeration areas; (2) the change trend of the AQI value also has obvious spatial autocorrelation, and generally presents a downward trend. However, the AQI value in a small number of regions, such as Xinjiang, shows a slow decline or even a reverse rise; (3) there are some of the main factors affecting AQI, such as GDP per capita, percentage of forest cover, total emissions of SO_2_, and these factors have different impacts on different regions. In addition, the increase of GDP per capita, the reduction of industrialization level, and the increase of forest coverage will significantly improve the air quality of other surrounding provinces. An in-depth analysis of the spatio-temporal evolution, influencing factors, and spillover effects of AQI in China is conducive to formulating countermeasures to improve air quality according to local conditions and promoting regional sustainable development.

## 1. Introduction

Since the reform and opening up, China’s industrialization and urbanization process has been accelerated unprecedentedly. With the sharp increase in the consumption of oil, coal, and other resources, China’s air pollution problem has gradually emerged [1,2,3]. In particular, many chemical factories often emit SO_2_, NO_2_, NO, and other harmful gases, causing serious pollution to urban air. Furthermore, in order to save production costs, low-grade chemical materials are used to discharge waste gas with excessive pollution into the atmosphere [4,5,6]. In January 2013, excessive haze that might be the worst air pollution incident in China since the last century hit China, affecting more than 8 million citizens [7]. This air pollution accident poses a huge threat to the life safety of citizens [8,9]. On 16 December 2016, the blue sky over Beijing was shrouded in a thick haze. On 21 December, 27 cities in Beijing, Tianjin, Hebei, and surrounding areas launched a red alert and 18 cities had launched an orange alert. On 26 November 2018, many places in Beijing, Tianjin, and Hebei were shrouded in gray haze. According to statistics, when the haze was serious, Beijing citizens would live in haze for nearly 1/3 of the days of the year approximately [10]. In addition, other surrounding provinces also have serious air pollution problems.

Although China’s economic growth has always been in the forefront of the world since the reform and opening up, behind the rapid growth China is also facing more serious environmental pollution problems, of which air pollution is one. It is not sustainable to rely solely on the economic growth brought about by high-speed industrialization and urbanization. It is not sustainable to trade for high-speed economic growth at the expense of the ecological environment. It is not sustainable to only pay attention to the immediate benefits of high-speed GDP growth while ignoring the long-term benefits of protecting the ecological environment [11]. After seeing the emergence of various ecological and environmental problems, Chinese leaders have decisively taken a series of measures [12,13,14]. With China’s emphasis on environmental pollution and increased investment in governance, the environment and ecology in various parts of China are getting better, and the air pollution problem in many areas is also gradually improving. People’s attention to air pollution has also promoted the diversification of air quality monitoring, measurement, and calculation methods. For example, Baldelli, A. (2021) used a new, low-cost, and reproducible platform to evaluate a low-cost multi-channel monitor for indoor air quality [15]. Popovicheva, O. et al. (2019) analyzed the microstructure and chemical composition of particles from small-scale gas flaring [16]. Penza, M. (2020) studied low-cost sensors for outdoor air quality monitoring [17]. Bulot, F.M. et al. (2019) carried out a long-term field comparison of multiple low-cost particulate matter sensors in an outdoor urban environment [18]. Although there are various methods for air monitoring and measurement, Air Quality Index (AQI) is the most typical indicator for measuring air quality and is well known.

The Air Quality Index (AQI) is an important indicator that can comprehensively reflect the quality of the air in a region. If the index is larger, it indicates that the local air quality is worse, and there are more pollutants in the air. On the contrary, if the index is smaller, the local air quality is better and there are fewer pollutants in the air. The calculation of this indicator takes into account many major pollutants such as fine particles (PM_2.5_), coarse particulate matter (PM_10_), sulfur dioxide (SO_2_), nitrogen dioxide (NO_2_), ozone (O_3_), and carbon monoxide (CO), which can comprehensively measure the air quality of a region [19,20,21,22]. However, what is the air quality situation in various regions of China? Which areas have serious air quality problems? As the Chinese government continues to attach importance to the environment, has the air pollution problem in various regions of China improved? How much are the specific effects? What factors are affecting the air quality in a region? To answer these questions, an in-depth analysis of air quality in various regions of China is needed. An in-depth analysis of the temporal and spatial evolution of the AQI and its influencing factors in various regions of China is helpful to the scientific formulation of air pollution policies in various regions of China and the realization of the goal of sustainable development.

At present, there are many studies on the indirect use of air quality index, such as Thanvisithpon, N. et al. (2021) [23], who built Air Pollution Adaptive Capacity (APAC) indicators based on the AQI level, and Zakaria et al. (2019) [24], who predicted the Air Pollution Index (API) indicators of Miri and Sarawak. It is worth mentioning that many scholars have analyzed the emission of a single pollutant. For example, Guo et al. (2022) [25] analyzed the spatio-temporal evolution and influencing factors of sulfur dioxide emissions in the Yangtze River Economic Belt of China from 1997 to 2017. Zhang et al. (2022) [26] analyzed spatio-temporal variation and influencing factors of TSP and anions in the coastal atmosphere of Zhanjiang City. In addition, direct analysis of the spatio-temporal evolution of the Air Quality Index (AQI) has gradually emerged in recent years. Although many scholars have analyzed the Air Quality Index status in China, their analysis focuses on the provincial data or cities in a province, for example, Yuan et al. (2019) [27] took the Beijing-Tianjin-Hebei region as an example to analyze the impact of China’s Air Pollution Control Policy on AQI; Yan et al. (2019) [28] analyzed the air quality in Shanghai; Wu et al. (2021) [29] analyzed the change trend of Air Quality Index in Shandong Province of China; Liu et al. (2016) [30] analyzed the air quality in Dalian and calculated the concentration of each pollutant; and Li et al. (2016) [31] calculated the AQI of 13 cities within the Beijing-Tianjin-Hebei region. Although some scholars tried to explore the temporal and spatial differentiation characteristics of air quality indexes across China, the data they collected were mainly limited to provinces and were difficult to refine. Qian et al. (2019) [32], Huang et al. (2018) [33], Liu et al. (2020) [34], and others use AQI data of various provinces in China. Liu et al. (2019) [35] analyzed the change of AQI in Chinese cities from 2014 to 2016. From the analysis of the results, most scholars generally concluded that China’s air quality is improving year by year. For example, Xiao et al. (2017) [36] found that the AQI of China over the past decade is improving year by year. Zhang et al. (2014) [37] analyzed the air quality in Wuhan and found that the AQI values in Wuhan showed a trend of gradual decline. From the perspective of the analysis method, the spatial analysis method is commonly used by most scholars. For example, Fang et al. (2015) [38] and He et al. (2017) [39] calculated Moran’s I of AQI and deeply analyzed its spatial autocorrelation. Yang et al. (2017) [40] found that the value of PM_2.5_ showed significant correlation along the Yangtze River economic belt. In general, most scholars generally believe that the air pollution degree in the study area has obvious spatial correlation characteristics.

From the above analysis, it can be seen that although the research on Air Quality Indexes in China has made rapid progress, due to data limitations, many scholars have only studied the spatial pattern of Air Quality Indexes in China’s provinces (municipalities, autonomous regions), and few scholars have refined the research on Air Quality Indexes to each county (city, district), and discussed the spatial differences and spatio-temporal evolution of AQI in counties as a unit. In addition, there is still a lack of research on the influencing factors and spillover effect of AQI. Many scholars have only studied the spatio-temporal evolution of the Air Quality Index, and obtained that the Air Quality Index generally shows a downward trend. However, they have not deeply explored what causes the decline of the Air Quality Index and what key factors will affect the local air quality.

After the reform and opening up, China’s economy has developed rapidly. However, environmental problems have gradually emerged along with economic development. Air pollution is one of the important problems. Understanding the current situation of air quality in China’s counties (cities, districts) and deeply discussing its influencing factors and spillover effect will help to better control air pollution and achieve the goal of sustainable development. However, it is still insufficient to discuss the spatial correlation of the Air Quality Index of each province in China roughly, which is not only unable to clearly grasp the air quality status of each county and city in the province, but also not conducive to in-depth discussion of various factors affecting air quality. For this reason, based on previous studies, this study further collected the Air Quality Indexes of various counties (including Hong Kong, Macao, and Taiwan) in China from 2014 to 2021, used cold and hot spot analysis, Moran’s I, and other spatial autocorrelation analysis methods to deeply analyze the spatio-temporal evolution of Air Quality Index in China, and analyzed its influencing factors based on provincial data. This study uses the spatial econometric model to deeply explore the causes behind the air pollution problem and its spillover effect, so as to provide reference for scientific treatment of air pollution and even for achieving sustainable development goals.

## 2. Materials and Methods

### 2.1. Research Method

#### 2.1.1. Spatial Autocorrelation Analysis

Many statistical data involve corresponding spatial locations and regions, and regions are often closely related, which causes many regional statistical data to have spatial correlation. The First Law of Geography shows that all things are related to other things, but things that are closer are more related than things that are far away [41]. With the development of science and technology, such as geographic information system (GIS), data containing regional space is gradually increasing. People pay more attention to the interrelation between regions. Especially, spatial factors need to be fully considered when analyzing the neighborhood effect, spillover effect, and peer effect. Therefore, spatial autocorrelation analysis came into being as the times require, and has gradually been widely valued. Before using spatial autocorrelation analysis, it is necessary to select an appropriate spatial weight matrix. Commonly used matrices include spatial adjacency weight matrix, spatial inverse distance weight matrix, and spatial economic distance matrix [42]. The methods for analyzing spatial autocorrelation include such as Moran’s I [43] and Geary’s C [44]. The Moran’s Index can be divided into Global Moran’s I and Local Moran’s I, and the calculation formula of Global Moran’s I is as follows:

(1) $$\mathrm{Global}\;\mathrm{Moran}’s\;I=\frac{ne^{T}We}{e^{T}e(\sum_{i}\sum_{j}w_{ij})}=\frac{\sum_{i}\sum_{j}w_{ij}(x_{i}-\bar{x})(y_{i}-\bar{y})}{S^{2}(\sum_{i}\sum_{j}w_{ij})}$$

where, ***e*** represents the residual matrix; ***W*** represents the spatial weight matrix; and *S*^2^ is the variance of observation *x_i_*. Although the variance expression of Global Moran’s I is relatively complex, which is recorded as Var(Global Moran’s I), it can be proved that the standardized Global Moran’s I follows a progressive normal distribution:

(2) $$\frac{\mathrm{Global}\;\mathrm{Moran}’s\;I-E\left(\mathrm{Global}\;\mathrm{Moran}’s\;I\right)}{\sqrt{\mathrm{Var}\left(\mathrm{Global}\;\mathrm{Moran}’s\;I\right)}}\overset{d}{\to }N\left(0,1\right)$$

Therefore, we can analyze whether the data has obvious spatial autocorrelation according to whether the statistics constructed in Formula (2) are significant. Although Global Moran’s I can clearly express the overall spatial autocorrelation, it is unable to analyze the spatial agglomeration of specific individuals or an area *i*, while Local Moran’s I can solve this problem. The calculation formula of Local Moran’s I is:

(3) $$\mathrm{Local}\;\mathrm{Moran}’s\;I=\frac{\left(x_{i}-\bar{x}\right)}{S^{2}}\cdot \sum_{j=1}^{n}w_{ij}(x_{j}-\bar{x})$$

The principle of Geary’s C is similar to that of Moran’s I, but because these two indexes cannot separate the “hot spot” and “cold spot” regions, so Getis and Ord (1992) [45] proposed Getis–Ord Gi*. The calculation formula of Getis–Ord Gi* is:

(4) $$\mathrm{Getis}-\mathrm{Ord}\;\mathrm{Gi}*=\frac{\sum_{i=1}^{n}\sum_{j=1}^{n}w_{ij}x_{i}x_{j}}{\sum_{i=1}^{n}\sum_{j\neq i}^{n}x_{i}x_{j}}$$

Similarly, the standardized Getis–Ord Gi* also follows the progressive normal distribution:

(5) $$\frac{\mathrm{Getis}-\mathrm{Ord}\;\mathrm{Gi}*-E\left(\mathrm{Getis}-\mathrm{Ord}\;\mathrm{Gi}*\right)}{\sqrt{\mathrm{Var}\left(\mathrm{Getis}-\mathrm{Ord}\;\mathrm{Gi}*\right)}}\overset{d}{\to }N\left(0,1\right)$$

Therefore, we can analyze whether the data have obvious spatial autocorrelation according to whether the statistics constructed in Formula (5) are significant. In addition, we can also analyze the Getis–Ord Gi* of an area *i*:

(6) $$\mathrm{Local}\;\mathrm{Getis}-\mathrm{Ord}\;\mathrm{Gi}*=\frac{\sum_{j\neq i}w_{ij}x_{j}}{\sum_{j\neq i}x_{j}}$$

If Moran’s I and Getis–Ord Gi* both show that the data have obvious spatial autocorrelation, spatial econometric methods can be used to control the spatial autocorrelation when analyzing the influencing factors to obtain more accurate estimation coefficients.

#### 2.1.2. Introduction of Spatial Econometric Model

Econometrics is widely used, not only in economics, but also in public management, geography, medicine, finance, and other fields. There are many examples in China and other countries [46]. The research methods of spatial econometrics have become increasingly mature in the past 10 years, and gradually become a frontier branch of econometrics [47,48,49].

**Figure 1:**
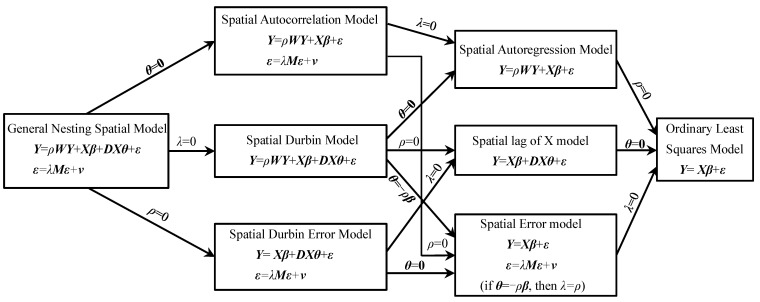
Relationship of various spatial econometric models.

The generalized spatial panel model can be expressed as [50]:

(7) $$\{\begin{array}{c} Y_{it}=\tau Y_{i,t-1}+\rho W_{i}Y_{t}+X_{it}\beta +D_{i}X\theta +u_{i}+\gamma_{t}+\varepsilon_{it}\\ \varepsilon_{it}=\lambda M_{i}\varepsilon_{t}+v_{it} \end{array}$$

where, *Y_i_*_,*t*−1_ represent the first order lag term of the dependent variable *Y_i_*_,*t*_ (i.e., it is a spatial dynamic panel model when *τ* ≠ 0, while *τ* = 0 is the spatial static panel model); this study selects the spatial static panel model for analysis. ***W****_i_*, ***D****_i_* and ***M****_i_*, respectively, represent the row of *i* of the spatial weight matrix ***W***, ***D*** and ***M***. ***X****_it_* represents the row of *i* of the independent variable matrix. ***β*** represents the parameter vector to be estimated. ***θ*** represents a fixed and unknown parameter vector to be estimated, *u_i_* stands for fixed effect and *γ_t_* stands for the time effect. *ε_it_* represents residual term. *ρ* and *λ* represents a spatial parameter. The Formula (7) is a General Nesting Spatial Model. However, it is complicated to use this model to estimate. This model can be simplified to other models under certain conditions. Figure 1 shows the relationship between various spatial econometric models [50].

As shown in Figure 1, when ***θ*** = **0**, the General Nesting Spatial Model (GNSM) can be converted to the Spatial Autocorrelation Model (SAC). If *ρ* = 0 or *λ* = 0, the SAC model can be converted to the Spatial Error Model (SEM) or Spatial Autoregression Model (SAR), respectively. When *λ* = 0, the GNSM can be converted to the Spatial Durbin Model (SDM). If ***θ*** = **0**, *ρ* = 0 or ***θ*** = −*ρ**β***, SDM can be converted to SAR, Spatial Lag of X Model (SLX), or SEM, respectively. When *ρ* = 0, the GNSM can be converted to the Spatial Durbin Error Model (SDEM). If *λ* = 0 or ***θ*** = **0**, SDEM can be converted to SLX or SEM, respectively. Finally, if the GNSM satisfies ***θ*** = **0** and *ρ* = *λ* = 0, it is the ordinary least squares model (OLS). In other words, OLS is actually the most special form of GNSM.

**Figure 2:**
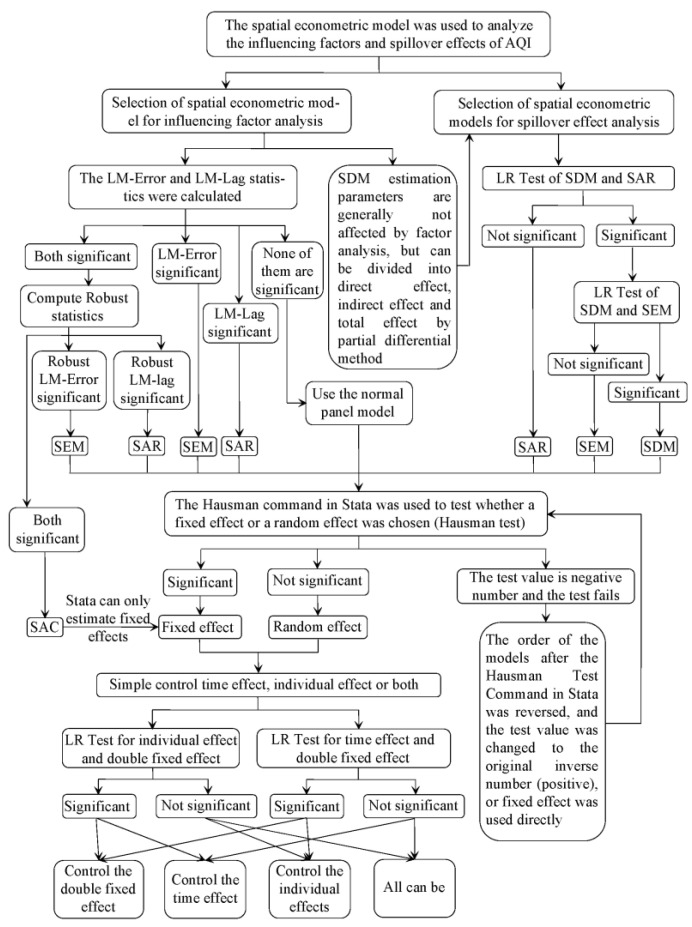
Tests of spatial econometric models and the selection of optimal model.

Although Figure 1 reveals the relationship between various spatial econometric models, the above models are complex and diverse. SDEM and SLX are not commonly used models. In addition, when selecting the optimal model, it is necessary to consider whether to control individual effects and time effects, and whether to use fixed effects or random effects. Therefore, in practical operation, it is often necessary to do some tests on various parameters and model settings to obtain the most appropriate model. Figure 2 shows the relevant steps of using Stata software to analyze the influencing factors and spillover effects of the AQI.

As shown in Figure 2, when analyzing the influencing factors of the AQI, the optimal model should be determined according to LM-Error, Robust LM-Error, LM-Lag, Robust LM-Lag, and other statistics, and the Hausman Test and LR Test should be used to determine whether to use fixed effects or random effects, and whether to control individual effects or time effects, respectively. It is worth noting that since the Spatial Durbin Model (SDM) not only includes the spatial correlation and non-spatial correlation of the dependent variable but also includes the spatial correlation and non-spatial correlation of the independent variables, its estimated coefficients do not reflect all the effects of the dependent variable, so the estimated coefficients of the SDM are generally not analyzed for influencing factors, and their comprehensive effects can be decomposed into direct effects, indirect effects, and total effects, so it can be used for the analysis of spillover effects. When analyzing spillover effects, it is necessary to determine whether to use SDM, SAR, or SEM according to the LR Test. Finally, according to the selected optimal model, the influencing factors and spillover effects are analyzed.

### 2.2. Data Selection

The dependent variable studied in this study is the Air Quality Index (AQI), which comes from the national urban air quality real-time release platform of the China National Environmental Monitoring Centre (http://www.cnemc.cn/), and data are updated daily (final accessed on 18 May 2022). This website provides hourly real-time air quality data from more than 2000 sites since May 2014. IDW (inverse distance weighting) is a common and simple spatial interpolation method. It uses the distance between the interpolation point and the sample point as the weight for the weighted average. The closer the sample point is to the interpolation point, the greater the weight will be given. IDW obtains interpolation units by averaging the values of each sampling point in the adjacent area. This method requires uniform distribution of discrete points, and the density is sufficient to reflect local surface changes in the analysis. This study uses the IDW (inverse distance weighting) method to interpolate the AQI data provided by the website into grid data (the resolution ratio is 0.1° × 0.1°), and then divides it into provinces, cities, and county-level regions (this study uses the administrative divisions of China in 2020) and conducts the average calculation. When analyzing the spatio-temporal evolution of the Air Quality Index, this study selects county-level regions from 2014 to 2021 to study. When analyzing the influencing factors of AQI, due to the limitation of Statistical Yearbook data collection, it is difficult to refine the data to the county level or city level, so this study collects the economic development, social population, natural environment, environmental governance and environmental pollution dimensions. Based on this, this study selects the AQI of 31 provinces (autonomous regions, municipalities directly under the Central Government) in China from 2014 to 2020 as the dependent variable when analyzing the influencing factors. With reference to the research of Zhang et al. (2022) [26], Yang et al. (2017) [40], Yang et al. (2021) [46], Yang et al. (2021) [50], Kuznets (1955) [51], Xu et al. (2019) [52], and Qin et al. (2020) [53], the indicator system of influencing factors of AQI is shown in Table 1.

The independent variable of this study selects five dimensions of panel data, including economic development, social population, natural environment, environmental governance, and environmental pollution. The data come from the EPS data platform. In order to maintain the stability of the data, this study takes the natural logarithm of all independent variables and AQI whose unit is not the percentage. The reason for taking the natural logarithm is to maintain the stability of the data. Before taking econometric model analysis, it is necessary to check the stability of the data; otherwise, it is easy to generate pseudo regression problems. If the data are unstable or do not have cointegration, they will draw a conclusion that might not convincing. Especially for time series data, it is necessary to make the data stable. Even for panel data, a series of methods to enhance the stability of the data is still needed, and taking the natural logarithm is one of the very simple and important methods. Furthermore, taking the natural logarithm has other advantages: (1) It will reduce the absolute value of data for easy calculation; (2) taking the natural logarithm can eliminate the multicollinearity and heteroscedasticity of data to a certain extent, making the estimation results of econometric models more reliable; (3) after taking the natural logarithm, the regression coefficient represents elasticity, that is, the estimated regression coefficient represents the change ratio of independent variable to dependent variable for every 1% change.

## 3. Results

### 3.1. Analysis of Spatial Pattern of AQI

**Figure 3:**
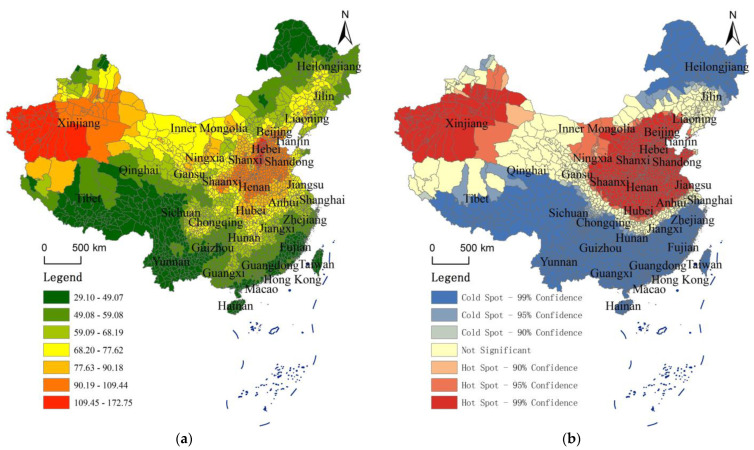
Distribution Pattern and Analysis Results of Getis–Ord Gi* of Air Quality Index (AQI) Based on the Levels of County. (**a**) Average AQI distribution of counties in China from 2014 to 2021; (**b**) analysis of cold and hot spots of average AQI of counties in China from 2014 to 2021.

Through the review of the literature, it can be found that many existing studies have not refined the Air Quality Index (AQI) to the county level. For this reason, this study collected data from more than 2000 sites and used the IDW spatial interpolation method to obtain the Air Quality Index (AQI) data of counties nationwide from 2014 to 2021. In order to better analyze the spatial pattern of AQI, this study averaged the AQI data of each county in China from 2014 to 2021, and obtained the average Air Quality Index of each county in the last 8 years. ArcGIS software was used to draw the distribution map of the Air Quality Index of each county in China, and the AQI was divided into several levels by using the natural breakpoint method. Different levels of regions have different colors on the map. The closer the area to red, the higher the Air Quality Index is (the worse the air quality is). The closer the area is to green, the lower the Air Quality Index is (the better the air quality is). In addition, this study constructs a spatial inverse distance weight matrix, uses ArcGIS software to calculate the Local Getis–Ord Gi* of each county, and conducts cold and hot spot analysis. See Figure 3 for all analysis results.

Figure 3 shows the average spatial distribution of AQI and the analysis results of cold and hot spots in each county from 2014 to 2021, which are detailed layer by layer, clearly showing the spatial differentiation characteristic of air quality in each region.

With reference to government websites and professional division (such as http://meeb.sz.gov.cn/hdjl/ywzsk/ggfwl/content/post_2027105.html and http://www.huizhou.gov.cn/zmhd/zczx/sthj/content/post_4686128.html, final access on 18 October 2022), the Air Quality Index (AQI) can be divided into six levels: grade I (AQI value between 0 and 50) indicates excellent air quality; grade II (AQI value between 51 and 100) indicates good air quality; grade III (AQI value between 101 and 150) indicates that there is slight air pollution; grade IV (AQI value between 151 and 200) indicates that there is moderate air pollution; grade V (AQI value between 201 and 300) indicates that there is heavy air pollution; grade VI (AQI value between 301 and 500) indicates that the air has been seriously polluted.

From the distribution map, the air quality of most provinces in the middle east and most counties in the northwest of China is generally poor, especially in the Hebei Province and most counties in Xinjiang. The analysis of cold and hot spots shows that the hot spots of AQI values in China are mainly divided into two regions, one is the region centered in Xinjiang, and the other is the region centered in Beijing, Tianjin, and Hebei. According to the map by county, the AQI values of many counties (cities and districts) in Hebei Province are 109.45~172.75 and the air pollution is extremely serious. The air quality of many counties and prefecture level cities near Hebei is also generally poor. The AQI values show a decreasing trend from the center of Hebei to the surrounding counties (cities and districts), but the decreasing trend is unknown. In particular, the AQI value of most areas to the south of Hebei is still high. The AQI value of most counties (cities and districts) in Shaanxi, Shanxi, Henan, and Shandong is still 90.19~109.44 and the air quality is still very worrying. From the perspective of northwest China, the air quality of most counties in Xinjiang is generally poor, and its distribution roughly shows the law of decreasing in the west of Xinjiang (AQI values are mainly distributed between 109.45 and 172.75) → the middle east of Xinjiang (AQI values are mainly distributed between 77.63 and 109.44) → the north of Xinjiang (AQI values are mainly distributed between 29.10 and 77.62), In addition, a few counties (cities and districts) in the northwest of Tibet are also affected by the western region of Xinjiang and show relatively serious air pollution (AQI values mainly range from 77.63 to 90.18). It is worth noting that the longitude of the most westerly and easterly crossing in Inner Mongolia is the largest, and the AQI values of various counties and cities in Inner Mongolia also have obvious differences, showing a decreasing trend from the west (AQI values are mainly distributed between 68.20 and 77.62) to the east (AQI values are mainly distributed between 29.10 and 68.19).

From the distribution map, the air quality in most provinces in the south of China and most counties in the northeast are generally great, especially in Yunnan, Tibet, Fujian, and some counties in the northeast. The analysis of cold and hot spots shows that the cold spots of AQI values in all parts of China are mainly divided into two regions, one is the regions in the south and southwest of China, and the other is the regions in the northeast of China (Heilongjiang and northeast of Inner Mongolia). According to the Figure 3, the regions with good air quality in China are mainly concentrated in the south and northeast of China. Most regions of Tibet, the west of Sichuan, Yunnan, Fujian, Hainan, Taiwan, the southeast of Guangdong, Hong Kong, Macao, the north of Heilongjiang, the northeast of Inner Mongolia, and other regions have the best air quality, and their AQI values are mainly distributed between 29.10 and 49.07. In addition, air quality in Qinghai, other regions of Heilongjiang, Guangxi, Zhejiang, other regions of Guangdong, Guizhou, and other regions are the second best, with the AQI value mainly distributed between 49.08 and 59.08.

The above analysis shows that China’s air pollution cities are mainly concentrated in the Beijing-Tianjin-Hebei region and its surrounding provinces, and most of Xinjiang is also have air pollution. The reasons for the two air pollution concentrations may be different. For the Beijing-Tianjin-Hebei region, the air pollution is mainly caused by industrialization. The secondary industry in these regions accounts for a large proportion, and heavy industry is developed, especially in the Hebei Province, which is heavily dependent on industry. The exhaust gas, waste residue, and inhalable particles from many factories cause serious pollution to the local air, and the polluted air will then spread to the surrounding areas. Therefore, different degrees of pollution also exist in various regions around the Beijing-Tianjin-Hebei region. It shows obvious characteristics of spatial agglomeration. The air quality in most areas of Xinjiang is mainly due to the fact that there are many barren mountains and wastelands, and few trees are planted.

The above analysis describes the air quality situation in China in more detail, but it mainly analyzes the average value of AQI, unable to compare the changes in the spatial pattern of each year. Therefore, this study uses ArcGIS software to calculate Global Moran’s I (Table 2) from 2014 to 2021, so as to further analyze the change of its spatial agglomeration.

It can be seen from Table 2 that the Z-statistics corresponding to Global Moran’s I have passed the 1% double tailed test, from the provincial dimension, the city dimension and county dimension, the *p* values are all less than 0.001, indicating that AQI has a very obvious spatial clustering feature. This is also in line with the actual law, because for the provinces with air pollution, the concentrations of NO, NO_2_, CO, SO_2_, and other gases are high, and these higher concentrations of polluting gases will spread to other areas along with the wind direction, especially the surrounding regions. The closer the areas are, the greater the impact will be. Therefore, many mathematicians have found that the AQI value has a very obvious spatial clustering feature.

### 3.2. Analysis of Change Trend of AQI

**Figure 4:**
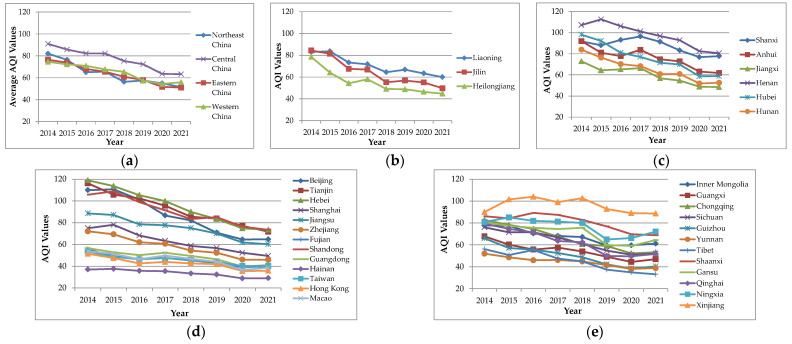
Change trend of AQI in each province from 2014 to 2021. (**a**) Change trend of average AQI values in northeast China, central China, eastern China, and western China; (**b**) change trend of AQI values of each province in northeast China; (**c**) change trend of AQI values of each province in central China; (**d**) change trend of AQI values of each province in eastern China; (**e**) change trend of AQI values of each province in western China.

Drawing the average AQI distribution map of each county from 2014 to 2021 and analyzing the cold and hot spots can clearly reveal the spatial pattern of the quality of air in various parts of China. However, the change trend of the AQI value in various regions from 2014 to 2021 cannot be seen. Therefore, this study draws the change trend map of the AQI in various provinces from 2014 to 2021 (Figure 4).

It can be seen from Figure 4 that the AQI values of most provinces from 2014 to 2021 will generally show a downward trend, especially in Beijing, Tianjin, and Hebei, and also in Shandong, Hubei, Hunan, Jilin, and other provinces. In addition, the AQI values of Yunnan, Tibet, Hong Kong, Macao, Taiwan, Hainan, and other places are also declining. Due to the good air quality and low AQI values, the trends are small. However, the AQI value in Xinjiang, where the air pollution is relatively serious, does not decline significantly. It is generally stable from 2014 to 2021, accompanied by signs of small fluctuations. In general, the air quality of most provinces located in and around Beijing, Tianjin, and Hebei is poor, but it declined significantly from 2014 to 2021, indicating that the local government had begun to focus on air pollution and take greater efforts to control it, while Xinjiang, which has poor air quality, has not improved significantly.

**Figure 5:**
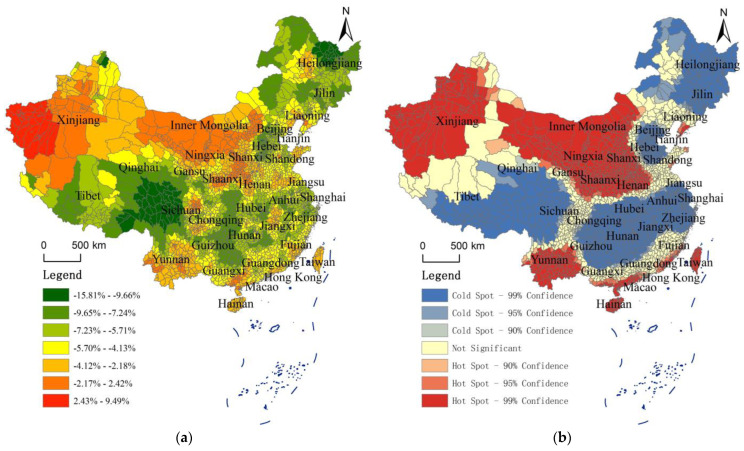
Annual average change rate of AQI in each county from 2014 to 2021 and analysis of its hot and cold spots. (**a**) Annual average change rate of AQI in each county from 2014 to 2021; (**b**) analysis of hot and cold spots of annual average change rate of AQI in each county from 2014 to 2021.

The above analysis can better reveal the change trend of the AQI value of each province from 2014 to 2021, but it is slightly rough only from the perspective of provinces, and it is difficult to see the spatial distribution characteristics of the decline of the AQI value more clearly. Therefore, the annual average change rate of the AQI in each county from 2014 to 2021 is calculated in this study, and the hot and cold spots are analyzed (Figure 5).

Figure 5 well reveals the spatial pattern characteristics of the change trend of the AQI value of each county from 2014 to 2021. It can be seen from Figure 5 that the AQI value in most regions has declined significantly from 2014 to 2021, with the fastest annual decline rate of 15.81%. The provinces with the fastest decline rate are mainly distributed in Tibet, Heilongjiang, Beijing–Tianjin–Hebei Region, Guizhou, Chongqing, and other places.

In some provinces, the air quality is poor but has improved significantly in recent years. For example, Beijing-Tianjin-Hebei is a region with serious air pollution, but fortunately, its AQI value has declined rapidly, which may be closely related to the government’s investment in environmental protection, which is a good sign. Some provinces have good air quality, but in recent years, the decline of their AQI values is relatively small. For example, Yunnan, Hainan, Taiwan, Guangxi, Guangdong, and other southern regions of China, although the decline of their AQI values is small, their air quality is generally great, and their AQI values are generally low, so the decline is small and reasonable, and there is no need to worry too much. Other provinces have good air quality and their AQI values have also declined significantly, such as Tibet, Heilongjiang, Guizhou, etc. This may be due to the fact that the local places pay more attention to the environment and invest more, which is a very fortunate result.

However, there are also many regions with poor air quality, but in recent years there is a trend of deterioration rather than improvement. For example, Xinjiang itself has relatively serious air pollution. In recent years, its AQI value has increased rather than decreased in some counties, and the air quality has shown a trend of deterioration. In addition, central and western Inner Mongolia, Ningxia, Shaanxi, Shanxi, Henan, and other places had high AQI values, but the AQI value did not decline significantly from 2014 to 2021, or even rose instead of falling. The air quality problem in these areas is very worrying. The local government needs to pay attention to it, enhance the awareness of environmental protection, and invest heavily in air pollution treatment.

### 3.3. Analysis of Influencing Factors of AQI

#### 3.3.1. Model Test Results and Selection

The results of spatio-temporal evolution analysis show that there is a significant spatial correlation feature in air quality index in China, and only using the general panel model may lead to a certain deviation in the estimation results and affect the conclusions. However, when using the econometric model, adding various explanatory variables may also slow down its spatial autocorrelation, so that the model does not have spatial correlation and can obtain more accurate estimation coefficients and standard errors. Is that the case? If the independent variables are added and the traditional OLS model is adopted, can the spatial autocorrelation be completely eliminated? This requires a spatial autocorrelation test on the regression results of the OLS model. Table 3 shows the test results after adding all explanatory variables and removing multicollinearity variables.

As shown in Table 3, Moran’s I and Robust Lagrange Multiplier in the test results of spatial error terms are not significant (failed to pass the 5% significance level test) whether or not multiple collinear variables are excluded, indicating that there is no obvious spatial autocorrelation problem in the spatial error terms. However, all test results of spatial lag terms pass the 1% significance level test, and the P value is less than 0.001. It shows that there is a very obvious spatial autocorrelation problem in the spatial lag term, and it is necessary to use the spatial econometric model to control the spatial lag term to obtain a more accurate estimate, while the spatial error term does not need to be controlled because it is not significant. According to the preliminary test results in Table 3, the SAR model can be selected to control the spatial lag term, and the SAR model will be the optimal model for analyzing the influencing factors. However, the choice of fixed effect or random effect, whether to control an individual or time effect still needs to be determined according to the actual test results, and when analyzing its spillover effect, it also needs to determine the appropriate model according to the LR test.

#### 3.3.2. Model Estimation Results and Analysis

The air quality in different parts of China is quite different, and has obvious spatial correlation characteristics. However, it is not enough to analyze the spatial correlation characteristics only, because we cannot clearly understand what causes the air quality differences in different parts of China, so it is necessary to analyze the influencing factors of AQI. In this study, the OLS model is built according to the variables of different dimensions in Table 1. Because of the collinearity, two variables including “Urban Population Density” and “Total emissions of NOx” are excluded. However, air quality in China has obvious spatial correlation characteristics. The spatial autocorrelation test results of the OLS model also show that the model has obvious spatial autocorrelation. After analysis, the SAR model needs to be used as the optimal spatial econometric model to better control the spatial autocorrelation.

Although the results of the spatial autocorrelation test show that using the SAR model is better, this study shows the results of various spatial econometric models (Table 4). In this way, we can not only compare the test results to further verify that the SAR model is the best estimation model, but also judge whether the model is robust by the difference of the estimation results of each model. Therefore, it is necessary to verify the robustness of models. In addition, in the spatial pattern analysis, this study found that the air pollution problems in Xinjiang, the Beijing-Tianjin-Hebei region, and other regions may not be caused by the same factor. Although the spatial autocorrelation problem can be solved by using the spatial econometric model, it is still necessary to classify different regions in the analysis of influencing factors, because the influencing factors of air quality indexes in different categories of regions may be inconsistent. Considering the problem of insufficient sample size caused by too fine division of regions, this study divides China into two regions: one is the provinces in the east and northeast of China, and the other is the provinces in the west and middle of China. Through the use of the SAR model, this study conducts a study on the influencing factors of zoning. Among them, the SAR-1 model includes all provinces in China, the SAR-2 model includes provinces in eastern and northeastern China, and the SAR-3 model includes provinces in western and central China. Finally, in order to enhance the robustness of the model, robust standard error estimation is used for all estimation results. The estimated results of all models are shown in Table 4.

Neither the traditional OLS model nor the panel model can be used to estimate the variables with obvious spatial autocorrelation, so the estimation results of the fix effect model may have some deviation. This study uses the spatial econometric model to estimate, and presents the estimation results of the SAC, SDM, SEM, and SAR models (Table 4). As shown in Table 4, when using the SAC model to estimate the parameters *ρ*, the estimation result has passed the 1% significance level test. While the estimation result of the parameter λ has failed to pass the 10% significance level test, indicating that there is no obvious spatial autocorrelation problem in the spatial error term, so SEM is not suitable, and the SAC model can also be further simplified to the SAR model. The significant level of estimation results of parameter *ρ* and parameters λ are similar to those in Table 3. They all show that the SAR model is more suitable, because their test results show that only the interference from the spatial lag term needs to be controlled, while the spatial error term does not have obvious spatial autocorrelation problems, so it is not necessary to control the interference from the spatial error term.

In this study, the Hausman Test and LR Test are used to select the appropriate SAR model. The test results show that, considering all provinces in the country, the statistics of the Hausman Test of the SAR-1 model failed to pass the 5% significance level test, so the random effect is better. The samples of the SAR-2 model are from eastern and northeastern provinces of China and the statistics of the Hausman Test passed the 1% significance level test, so the fixed effect model is better. The samples of the SAR-3 model are all provinces in central and western China, and the statistics have not passed the 5% significance level test, so it is better to use random effects. The LR test statistics of the time effect of the SAR-2 model passed the 1% significance level test, indicating that it should be significant differences between simple control time effect and control double effect, and individual effect should be considered. The LR test statistics of individual effects failed to pass the 5% significance level test, indicating that there was no significant difference between controlling individual effects and controlling double effects, and time effects could not be controlled. Therefore, this study will chose SAR-2 model that only control individual effects. In the SAR-1, SAR-2, and SAR-3 models, the seven indicators of per capita GDP, industrialization level, population density, percentage of forest cover, total emissions of SO_2_, green space rate of built-up areas, and sewage treatment rate are relatively significant, indicating that they are important factors affecting AQI. So this study will conduct in-depth analysis on these important indicators.

(1) Per capita GDP. Based on the national data, the estimated results of the SAR-1 (RE) model passed the 5% significance level test, with an estimated coefficient of −0.1326, which means that when other conditions remain unchanged and the impact of spatial lag is considered, the Air Quality Index of each region will decline by 0.1326% for every 1% increase in per capita GDP. Although the estimation results of the SAR-2 (FE) and SAR-3 (RE) models are not significant, their estimation coefficients are both negative, and the absolute value of SAR-2 (FE) estimation coefficients is larger. In addition, the estimation results of all other models are negative, and the difference of estimation coefficients is small, indicating that the model is robust. The above results show that the increase of per capita GDP can promote the improvement of local air quality, and the per capita GDP has a greater impact on the AQI of eastern and northeastern provinces of China. That is to say, the growth of GDP per capita has not worsened the air quality, but has promoted the improvement of air quality. It can also be seen that as people become richer and the economy grows rapidly, China is paying more attention to the problem of air pollution. Since 2014, China has not sacrificed the ecological environment too much for rapid economic growth. With economic growth, the air quality in various parts of China is improving. This is more obvious in eastern provinces of China.

(2) Industrialization level. Based on the national data, the estimated results of the SAR-1 (RE) model passed the 1% significance level test, and its estimated coefficient was 0.0086, which means that when other conditions remain unchanged and the impact of spatial lag is considered, the Air Quality Index of each region will increase by 0.0086% for every 1% increase in the output value of the secondary industry. The estimation results of the SAR-2 (FE) and SAR-3 (RE) models are also very similar, and the absolute value of the estimation coefficient of SAR-2 (FE) is larger. In addition, the estimation results of all other models are positive, the difference of estimation coefficients is small, and most of them are very significant, indicating that the model is robust. The above results show that the increase of the output value of the secondary industry will make the local air quality face a greater threat of pollution, and this threat is more obvious in eastern and northeastern China. This is because some provinces and regions in the east and northeast of China have developed heavy industry and rely heavily on industrialization, which is typical of the Hebei Province. The consequence is that a large number of polluting gases are emitted, which worsens the local air quality. This also further enlightens us that we need to further change the development mode of these regions, take the road of green development, achieve high-quality development, and do not sacrifice the environment for the sake of industrialization.

(3) Population density. Based on the national data, the estimated results of the SAR-1 (RE) model passed the 1% significance level test, and its estimated coefficient was 0.1030, which means that when other conditions remain unchanged and the impact of spatial lag is considered, the Air Quality Index of each region will increase by 0.1030% for every 1% increase in population density. However, the estimation results of the SAR-2 (FE) and SAR-3 (RE) models are significantly different: the estimation result of SAR-3 (RE) is 0.1208, and the estimation result of SAR-2 (FE) is −0.2863. The above results show that for the central and western provinces of China, the increase of population density leads to the deterioration of air quality, while for the eastern and northeastern provinces of China, the increase of population density is beneficial to improve air quality. The reason for this seemingly “contradictory” result may be related to the local people’s awareness of environmental protection and population mobility. The population demarcation line shows that the population density in the southeast of China is relatively high, while that in the northwest is relatively low. In addition, most provinces and regions in the east of China, especially in the southeast coastal areas, are economically developed, and people have a relatively strong awareness of environmental protection. In addition, the net inflow of people in the east is relatively large, so more and more concentrated people need a fresh air environment, which further forces the local people to strengthen environmental protection and reduce the emission of air pollutants. For example, Beijing, as the capital of China, has experienced smog many times, but its population density is very high. With the continuous migration of the population and the growing demand for environmental protection, Beijing has paid more attention to environmental protection in recent years. The local government has also issued a number of measures, and the AQI has declined significantly. On the contrary, the economic level of most provinces in central and western China is weaker than that of southeast coastal areas, and they pay less attention to environmental protection. In addition, most regions in central and western China have fragile economy and harsh environment, so the increase of population may cause air pollution. This is because: (a) many regions in central and western China have poor natural conditions, for example, some regions show obvious karst rocky desertification characteristics, some regions are short of resources, and some regions are seriously polluted. The excessive increase of population may lead to the intensification of the contradictions between population and natural resources, population and land in these areas, which may aggravate environmental pollution; (b) most of the central and western regions of China have made fewer efforts to improve the environment and air than the eastern regions of China, which is largely due to financial constraints and other aspects. In some other industrialized regions, such as Beijing, Tianjin, and Hebei, the funds used for environmental remediation are sufficient. With the increase of people’s calls for environmental restoration, the funds used for environmental governance and air pollution are very obvious. However, the economic, social, ecological, and other conditions in central and western China are relatively fragile, and the funds used for environmental quality are less than those in eastern China. With the increase of population, its air pollution will become more serious; (c) the air quality in some southwestern regions in central and western China such as Yunnan, Tibet, Guangxi, Sichuan are generally good, and these regions may pay relatively less attention to air pollution.

(4) Percentage of forest cover. Based on the national data, the estimated results of the SAR-1 (RE) model passed the 1% significance level test, and its estimated coefficient was −0.0077, which means that when other conditions remain unchanged and the impact of spatial lag is considered, the Air Quality Index of each region will decline 0.0077% for every 1% increase in forest coverage. The estimation results of the SAR-2 (FE) and SAR-3 (RE) models are also very similar, and the absolute value of the estimation coefficient of SAR-3 (FE) is larger. In addition, the estimation results of most other models (except the Spatial Durbin Model) are significant, and the estimation coefficients are negative, indicating that the model is robust. The above results show that the increase of forest coverage will promote the improvement of local air quality, which is more obvious in central and western China. This is because most provinces and regions in central and western China have relatively poor natural conditions, with high elevations and slopes, especially in Yunnan Province [54,55,56]. In addition, there are many barren mountains and wastelands in Xinjiang. The natural conditions are extremely poor, and the forest coverage is very low, resulting in serious air pollution. The regression results of the model are robust and they show that the increase of forest coverage will significantly improve the air quality, which further enlightens us that Xinjiang and other heavily polluted areas need to plant more trees to improve the forest coverage. Even if it is not a seriously polluted province, it can improve the air quality by planting trees and strictly limiting the amount of wood cutting.

(5) Total emissions of SO_2_. Based on the national data, the estimated results of the SAR-1 (RE) model passed the 5% significance level test, with an estimated coefficient of 0.0252, which means that when other conditions remain unchanged and the impact of spatial lag is considered, the Air Quality Index of each region will increase by 0.0252% for every 1% increase in total sulfur dioxide emissions. The estimated result of the SAR-2 (FE) model is 0.0266, and it passed the 5% significance level test. The estimated results of the SAR-3 (FE) model are not significant. In addition, the estimation results of most other models (except the fixed effect model) are significant, and the estimation coefficients are positive, indicating that the model is robust. The above results show that the increase of emissions of SO_2_ will worsen the local air quality, which is more obvious in eastern and northeastern China. This is because some provinces in the east and northeast of China are more industrialized and emit more SO_2_ and other polluting gases, resulting in air pollution. It also further enlightens us that for some regions in eastern and northeastern China that rely on industrial development, relevant measures should be formulated in time to control the emission of SO_2_ and other polluting gases, so as to achieve energy conservation and emission reduction, and not to sacrifice the environment to save costs for petty profits.

(6) Green space rate of the built-up area. Based on the national data, the estimated results of the SAR-1 (RE) model passed the 5% significance level test, with an estimated coefficient of −0.0086, which means that when other conditions remain unchanged and the impact of spatial lag is considered, the Air Quality Index of each region will decrease by 0.0086% for every 1% increase in the green space rate of built-up areas. The estimation result of the SAR-2 (FE) model is not significant. The estimated result of the SAR-3 (FE) model is −0.0169, and it passed the 1% significance level test. In addition, the estimation results of most other models (except the fixed effect model and Spatial Durbin Model) are significant, and the estimation coefficients are negative, indicating that the model is robust. The above results show that the increase of green space rate in built-up areas improves the local air quality. This conclusion is similar to the previous conclusion that the increase of percentage of forest cover promotes the improvement of air quality, which further indicates that afforestation can effectively promote the improvement of air quality. Especially for most of the central and western provinces with poor environment, it is necessary to further increase the percentage of forest cover and green space rate to improve air quality.

(7) Sewage treatment rate. Based on the national data, the estimated results of the SAR-1 (RE) model passed the 1% significance level test, and its estimated coefficient was −0.0033, which means that when other conditions remain unchanged and the impact of spatial lag is considered, the Air Quality Index of each region will decrease 0.0033% for every 1% increase in sewage treatment rate. The estimation result of the SAR-2 (FE) model is not significant; the estimated result of the SAR-3 (FE) model is −0.0035, and it passed the 1% significance level test. In addition, the estimation results of most other models (except the fixed effect model) are significant, and the estimation coefficients are negative, indicating that the model is robust. The above results show that the increase of sewage treatment rate will improve the local air quality, which is more obvious in central and western China. This is because the sewage treatment rate can reflect the treatment capacity of a region for wastewater, waste gas, and solid waste. The treatment capacity of sewage is closely related to the treatment capacity of waste gas and solid waste. If the sewage treatment rate is relatively low, it indicates that the technological level of local waste treatment is relatively low, thus affecting the local air quality, especially for the regions in central and western China, as their economic conditions and scientific and technological level are generally weaker than those in the eastern region, and its treatment capacity for pollutants such as sewage and waste gas is lower, so the sewage treatment rate in central and western China has a more obvious impact on AQI.

In general, this study finds that the per capita GDP, industrialization level, population density, percentage of forest cover, total emissions of SO_2_, green space rate in built-up areas, and sewage treatment rate will have a relatively significant impact on the air quality of the regions. The increase of per capita GDP, percentage of forest cover, green space rate in built-up areas, and sewage treatment rate will promote the improvement of local air quality, the acceleration of industrialization level and the increase of total emissions of SO_2_ will lead to the deterioration of local air quality, and these factors have different impacts on different regions. For example, the impact of per capita GDP, industrialization level, and total emissions of SO_2_ is more obvious in the east and northeast of China, and the impact of forest coverage, green space rate in built-up areas, and sewage treatment rate is more obvious in central and western China. In addition, the increase of population density in eastern and northeastern China will promote the improvement of local air quality, while in central and western China it will have an inhibitory effect, which may be related to the local people’s awareness of environmental protection and population mobility.

### 3.4. Analysis of Spillover Effect of AQI

Although this study has deeply analyzed the impact of various factors on AQI, the above analysis is mainly limited to each province. Although the model has well controlled the interference of spatial autocorrelation and obtained more accurate estimation results, it is worth noting that due to the existence of spatial autocorrelation, the above factors will not only affect the air quality of the region, but also may affect the air quality of surrounding regions. Therefore, it is not enough to simply use the SAR model to analyze the influencing factors of AQI. We also need to analyze the impact of these important influencing factors on surrounding provinces, that is, to analyze the spillover effect of the important influencing factors of AQI. Although the estimated results of the Spatial Durbin Model cannot reflect its real effects, it is very practical in analyzing spillover effects. At the same time, the analysis of spillover effects can also be conducted using SAR, SEM or SDM, and the optimal model needs to be determined according to the LR test (Table 5).

As shown in Table 5, the statistics of the LR test of SDM and SAR and the LR test of SDM and SEM passed the 1% significance level test, and their P values were less than 0.01, indicating that the SDM model cannot be simplified into SAR or SEM when analyzing spillover effects. Therefore, this study intends to use the Spatial Durbin Model (SDM) to analyze the spillover effects of each core influencing factor. According to the test results in Table 4, this study selects random effects and uses the SDM to decompose the effects into direct effects, indirect effects (spillover effects), and total effects (Table 6). To save space, Table 6 only lists the effect decomposition results of factors that have a significant impact on AQI in the analysis of influencing factors.

As shown in Table 6, no matter the direct effects, indirect effects (spillover effects), or total effects, the estimated results are quite different from those of SDM (RE) in Table 4. In particular, the estimated coefficient of percentage of forest cover is not significant in SDM (RE) in Table 4. However, after using SDM (RE) and decomposing each effect, the direct effect, indirect effect (spillover effect) and total effect have passed the 5%, 1% and 1% significance level test respectively. This difference also further shows that the estimation coefficients made by using the SDM alone cannot reflect the influence of various factors on the dependent variable AQI very well. It is necessary to use the partial differential method to decompose them into various effects for in-depth research. From the decomposition results of the SDM, the spillover effects of per capita GDP, industrialization level, and percentage of forest cover are obvious, so this study intends to analyze the spillover effects of the above three factors.

(1) Per capita GDP. The analysis of influencing factors shows that the increase of per capita GDP can promote the improvement of local air quality, and the per capita GDP has a greater impact on AQI in eastern and northeastern provinces of China. Using the SDM to decompose the effects, it can be found that the per capita GDP has a small impact on the local air quality, but has a significant impact on other surrounding provinces. The estimated result of the spillover effect is −0.3392, and has passed the 1% significance level test. It shows that when other conditions remain unchanged, every 1% increase in the local per capita GDP will promote the AQI value of other surrounding provinces to decline 0.3392%. With economic growth, the local government has gradually attached importance to the problem of air pollution, which not only promotes the improvement of air quality in the province, but also drives the economic development of other surrounding provinces, and also promotes the enhancement of environmental awareness in the surrounding areas. In addition, AQI has a very obvious spatial agglomeration feature. With the improvement of local air quality, it will also promote the improvement of air quality in other surrounding provinces, thus producing an obvious spillover effect.

(2) Industrialization level. The analysis of influencing factors shows that the increase of the output value of the secondary industry will make the local air quality face a greater threat of pollution, and this threat is more obvious in eastern and northeastern China. Using SDM to decompose the effects, it can be found that the improvement of industrialization level not only seriously threatens the local air quality, but also has a significant impact on other surrounding provinces. The estimated result of spillover effect is 0.0139, and it passed the 1% significance level test. It shows that when other conditions remain unchanged, every 1% increase in the output value of the secondary industry will lead to an increase of the AQI value of other surrounding provinces by 0.0139%. This is because the AQI has a very obvious feature of spatial agglomeration. With the acceleration of local industrialization, the emissions of local pollutants increase significantly, which not only threatens the local air quality, but also may spread to other surrounding provinces, threatening the air quality of other regions. In addition, in consideration of costs, benefits and cooperation, industrial enterprises have obvious spatial agglomeration in site selection. The development of local industrialization will promote the development of industrialization in other surrounding provinces, resulting in a closer spatial agglomeration in the industrialization process (for example, the Beijing-Tianjin-Hebei region has a strong dependence on industry), which will further aggravate the air pollution problem in other regions.

(3) Percentage of forest cover. The analysis of influencing factors shows that the increase of percentage of forest cover will promote the improvement of local air quality, which is more obvious in central and western China. Using SDM to decompose the effects, it can be found that the increase of percentage of forest cover will not only improve the local air quality, but also promote the improvement of the air quality of surrounding provinces. The estimated result of the spillover effect is −0.0194, and it has passed the 1% significance level test. It shows that when other conditions remain unchanged, every 1% increase of percentage of forest cover will lead to the decrease of the AQI value of other surrounding provinces by 0.0194%. Many studies have shown that afforestation can not only improve the local environment, but also benefit other surrounding areas, with positive external effects. Therefore, increasing the local forest coverage is not only conducive to improving the local environment, but also can further improve the environmental quality of surrounding areas and promote regional sustainable development.

In general, the per capita GDP, industrialization level, and percentage of forest cover show obvious spillover effects. The increase of per capita GDP, the reduction of industrialization level, and the increase of percentage of forest cover will significantly improve the air quality of other surrounding provinces.

## 4. Conclusions and Discussion

### 4.1. Conclusions

After the reform and opening up, China’s economy has developed rapidly. However, environmental problems have gradually emerged along with economic development. Air pollution is one of the important problems. Understanding the current situation of air quality in China’s counties (cities, districts) and deeply discussing its influencing factors will help to better control air pollution and achieve the sustainable development goals. Although the research on the Air Quality Index (AQI) in China has made rapid progress, due to data limitations, many scholars have not refined the research on the AQI to every county (city, district), so as to further analyze the spatial differences of the AQI. In addition, there is still a lack of research on the influencing factors and spillover effects of the AQI. Many scholars have not deeply explored what causes the decline of the Air Quality Index and what key factors will affect the local air quality. In view of the lack of current research, based on previous studies, this study further collected the AQI of various counties (including Hong Kong, Macao, and Taiwan) in China from 2014 to 2021, used cold and hot spot analysis, Moran’s I, and other analysis methods to deeply analyze the spatio-temporal evolution of Air Quality Index in China, and analyzed its influencing factors based on provincial data; the spatial econometric model is used to deeply explore the causes behind the air pollution problem and deeply analyze the spillover effects, so as to provide reference for scientific treatment of air pollution and even for achieving sustainable development goals. The results show that:

(1) From 2014 to 2021, the AQI of all parts of the country showed obvious spatial agglomeration characteristics. From the map of counties, the air quality in most provinces in the middle east and most counties in the northwest of China are generally poor, especially in the Hebei Province and most counties in Xinjiang. The hot spots of AQI values in China are mainly divided into two regions, one is the region centered on Xinjiang, and the other is the region centered on Beijing–Tianjin–Hebei region. The cold spots of AQI values in China are mainly divided into two regions, one is most regions in the south and southwest of China, and the other is the northeast of China (Heilongjiang and northeast of Inner Mongolia).

(2) The analysis result of the time change trend of the AQI shows that the AQI value of most provinces will generally show a decrease trend from 2014 to 2021, especially in the Beijing-Tianjin-Hebei region, as well as Shandong, Hubei, Hunan, Jilin, and other provinces. However, the AQI value in Xinjiang, where the air pollution is relatively serious, does not decline significantly. It is generally stable from 2014 to 2021, accompanied by signs of small fluctuations. In addition, the change trend of the AQI value also has obvious spatial correlation. South of China, Xinjiang, central and western Inner Mongolia, Ningxia, Shaanxi, Shanxi, Henan, and other places belong to high-value clusters, while the Beijing-Tianjin-Hebei region, Heilongjiang, Tibet, and central and southern China belong to low-value clusters, with obvious spatial agglomeration. In particular, the air quality in some regions is poor, but in recent years it has not improved but has deteriorated, such as some counties in Xinjiang, central and western Inner Mongolia, Ningxia, Shaanxi, Shanxi, and Henan.

(3) The analysis results of influencing factors show that the per capita GDP, industrialization level, population density, percentage of forest cover, total emissions of SO_2_, green space ratio in built-up areas, and sewage treatment rate will have a relatively significant impact on the air quality of a region. The increase of per capita GDP, percentage of forest cover, green space ratio in built-up areas, and sewage treatment rate will promote the improvement of local air quality, the acceleration of industrialization level and the increase of total emissions of SO_2_ will lead to the deterioration of local air quality, and these factors have different impacts on different regions. For example, the impact of per capita GDP, industrialization level, and total emissions of SO_2_ is more obvious in the east and northeast of China, and the impact of percentage of forest cover, green space rate in built-up areas, and sewage treatment rate is more obvious in central and western China. In addition, the increase of population density in eastern and northeastern China will promote the improvement of local air quality, while in central and western China it will have an inhibitory effect, which may be related to the local people’s awareness of environmental protection and population mobility.

(4) The results of the spillover effect analysis show that the per capita GDP, industrialization level, and percentage of forest cover show obvious spillover effects. The increase of per capita GDP, the decrease of industrialization level, and the increase of percentage of forest cover will significantly improve the air quality of other surrounding provinces.

An in-depth analysis of the spatio-temporal evolution, influencing factors, and spillover effects of the AQI in China is conducive to formulating countermeasures to improve air quality according to local conditions and promoting regional sustainable development goals.

### 4.2. Discussion

When analyzing the spatio-temporal evolution of the AQI, this study found that although the air quality in most regions has improved significantly in recent years, especially in the Beijing-Tianjin-Hebei region, the AQI value has declined significantly. There are still some regions where the air pollution is still relatively serious, especially in many regions where the air quality is poor, and in recent years, the air quality has not improved but has also deteriorated. Although the local government has begun to pay more attention to environmental and ecological protection, the air quality problem in some areas is still very worrying. In recent years, the AQI value in some highly polluted areas has increased rather than decreased, which is also worth thinking deeply. For this reason, this study suggests that the Chinese government, while strengthening the environmental pollution treatment, incline the governance funds to Xinjiang, the central and western regions of Inner Mongolia, Ningxia, Shaanxi, Shanxi, Henan, and other regions where the AQI value does not decrease but increases, and at the same time incline to the counties (cities, districts) with serious air pollution in these provinces. For the local high pollution industries, more detailed pollutant emission measures should be further developed to prevent enterprises from damaging the environment in order to reduce costs and increase profits, and higher taxes should be levied on the high pollution industries to compensate for the negative external effects on the local environment, and promote energy conservation and emission reduction, and formulate more detailed environmental protection measures. We will improve the air quality monitoring mechanism in highly polluted areas, change the mode of local economic development, advocate green and sustainable development, and resolutely abandon the idea of sacrificing the environment for faster short-term economic growth.

In addition, according to the analysis results of the influencing factors of the AQI and spillover effect, this study suggests that all regions in China can formulate countermeasures to improve air quality according to local conditions. As far as the central and western regions of China are concerned, the altitude and slope are generally high, and the local natural conditions are worse than those in the east. Therefore, improving the local percentage of forest cover, green space ratio in built-up areas, and wastewater and waste gas treatment efficiency can more effectively promote the improvement of air quality, which is most typical of Xinjiang. In most areas of Xinjiang, there are many barren mountains and wastelands, and there are also many desert areas. The AQI value remains high for a long time. The local government should pay more attention to the environment, increase investment in environmental protection, plant trees in barren mountains and wastelands, and expand the local percentage of forest cover to effectively control the air pollution problem. In addition, the percentage of forest cover has a significant spillover effect, which can significantly promote the improvement of air quality in the surrounding areas. Therefore, the Chinese government can also implement a preferential policy of funds for high air pollution areas in the west, such as Xinjiang, to encourage local afforestation to improve the percentage of forest cover and green space rate, and make full use of its positive external effects. For the northeast and eastern regions, the impact of per capita GDP, industrialization level, total emissions of SO_2_, and other factors is more obvious, which further enlightens us: for most high pollution areas in eastern China, the pollutant emission standards of high pollution enterprises should be further refined and strictly enforced, environmental protection should be strengthened, and strict energy conservation and emission reduction measures should be formulated for high pollution enterprises. We will increase investment in pollution treatment and tackle air pollution from the source.

## Figures and Tables

**Table 1:** Index system of influencing factors of Air Quality Index (AQI).

Attribute	Dimension	Variable	Calculation Method or Data Description	Unit
Dependent Variable	Environment	Air Quality Index (AQI)	National Urban Air Quality Real-time Release Platform of China National Environmental Monitoring Centre	None
Independent Variable	Economic Development	Per Capita GDP	GDP/Total population	CNY/Person
		Industrialization Level	Output value of secondary industry/GDP × 100%	%
	Social Population	Urban Population Density	Total urban population/Urban land area	Person/km^2^
		Population Density	Total population/Total land area	Person/km^2^
		Population Urbanization Rate	Total urban population/Total regional population	%
	Natural Environment	Park Green Area Per Capita	Total area of park green space/Total population in the area	m^2^/Person
		Percentage of Forest Cover	Forest area/Total land area × 100%	%
		Green Space Rate of Built-up Area	Greening area of built-up area/Total area of built-up area × 100%	%
	Environmental Governance	Governance Efforts of Environmental Pollution	Total investment in governance of environmental pollution/GDP × 100%	%
		Governance Efforts of Industrial Waste Gas	Operation cost of industrial waste gas treatment facilities/GDP × 100%	%
		Governance Facilities of Industrial Waste Gas	Total number of industrial waste gas treatment facilities in the year	10,000 sets
		Sewage Treatment Rate	Sewage treatment capacity/total sewage discharge × 100%	%
		Domestic Garbage Harmless Treatment Rate	Amount of harmless urban domestic waste/total amount of urban domestic waste generated × 100%	%
	Environmental Pollution	Total emissions of SO_2_	Total SO_2_ emissions in the year	10,000 tons
		Total emissions of NO_x_	Total NO_x_ emissions in the year	10,000 tons

**Table 2:** Global Moran’s I Calculation Results from 2014 to 2021.

Dimension	Year	2014	2015	2016	2017	2018	2019	2020	2021	Annual Average
Province	Moran’s I	0.454	0.467	0.485	0.415	0.393	0.358	0.377	0.352	0.428
	Z-statistic	6.377	6.445	6.682	5.807	5.505	5.060	5.296	4.976	5.978
	*p* Value	0.000	0.000	0.000	0.000	0.000	0.000	0.000	0.000	0.000
City	Moran’s I	0.951	0.758	0.722	0.759	0.647	0.715	0.692	0.632	0.769
	Z-statistic	65.913	52.673	50.197	52.687	45.194	49.725	48.122	44.095	53.433
	*p* Value	0.000	0.000	0.000	0.000	0.000	0.000	0.000	0.000	0.000
County	Moran’s I	1.035	0.915	0.893	0.908	0.789	0.852	0.815	0.750	0.913
	Z-statistic	291.723	258.021	251.818	255.916	222.471	240.292	229.837	211.509	257.285
	*p* Value	0.000	0.000	0.000	0.000	0.000	0.000	0.000	0.000	0.000

**Table 3:** Spatial autocorrelation test results.

Items	Test Variable Name	Add All Independent Variable		Eliminate Independent Variables of Multicollinearity	
		Test Statistics	*p* Value	Test Statistics	*p* Value
Spatial Error	Moran’s I	0.780	0.435	0.796	0.426
	Lagrange Multiplier	3.761	0.052 *	4.430	0.035 **
	Robust Lagrange Multiplier	2.536	0.111	3.021	0.082 *
Spatial Lag	Lagrange Multiplier	18.293	0.000 ***	23.939	0.000 ***
	Robust Lagrange Multiplier	17.068	0.000 ***	22.530	0.000 ***

Note: *, **, ***, respectively, indicate that the original hypothesis is rejected at the significance level of 10%, 5%, and 1%.

**Table 4:** Estimation results of econometric model.

Variable and Test Result	Fix Effect	SAC(FE)	SDM(RE)	SEM(RE)	SAR-1(RE)	SAR-2(FE)	SAR-3(RE)
Per Capita GDP	−0.0410 (0.0527)	−0.0538 (0.0583)	−0.0585 (0.0479)	−0.0657 (0.0611)	−0.1326 ** (0.0544)	−0.0670 (0.0773)	−0.0488 (0.0832)
Industrialization Level	0.0050 * (0.0029)	0.0062 *** (0.0018)	0.0064 *** (0.0017)	0.0063 *** (0.0021)	0.0086 *** (0.0019)	0.0092 *** (0.0026)	0.0055 ** (0.0023)
Population Density	0.0006 (0.1710)	−0.0775 (0.1651)	0.0591 ** (0.0248)	0.0893 *** (0.0280)	0.1030 *** (0.0280)	−0.2863 ** (0.1410)	0.1208 *** (0.0350)
Park Green Area Per Capita	−0.0415 (0.1022)	−0.0040 (0.0648)	−0.0277 (0.0587)	−0.0332 (0.0622)	−0.0519 (0.0692)	0.1069 (0.0900)	−0.0840 (0.0838)
Population Urbanization Rate	−0.0033 (0.0124)	−0.0104 * (0.0056)	−0.0008 (0.0039)	−0.0036 (0.0047)	−0.0020 (0.0052)	−0.0056 (0.0115)	0.0171 (0.0129)
Population Urbanization Rate (Square term)	0.0000 (0.0001)	0.0001 * (0.0000)	0.0000 (0.0000)	0.0000 (0.0000)	0.0000 (0.0000)	0.0000 (0.0001)	−0.0005 * (0.0003)
Percentage of Forest Cover	−0.0089 * (0.0049)	−0.0074 * (0.0039)	−0.0018 (0.0017)	−0.0094 *** (0.0019)	−0.0077 *** (0.0020)	−0.0085 ** (0.0038)	−0.0090 *** (0.0025)
Governance Efforts of Environmental Pollution	0.0030 (0.0217)	0.0016 (0.0142)	0.0013 (0.0148)	0.0072 (0.0153)	0.0145 (0.0163)	0.0220 (0.0207)	−0.0012 (0.0172)
Governance Efforts of Industrial Waste Gas	−0.0293 (0.0373)	−0.0223 (0.0269)	−0.0171 (0.0258)	−0.0109 (0.0276)	−0.0376 (0.0305)	0.0861 (0.0798)	−0.0251 (0.0288)
Governance Facilities of Industrial Waste Gas	0.0210 (0.0309)	0.0277 (0.0188)	0.0459 *** (0.0169)	0.0328 (0.0204)	0.0043 (0.0179)	0.0329 (0.0207)	0.0282 (0.0232)
Total Emissions of SO_2_	0.0172 (0.0208)	0.0209 ** (0.0106)	0.0191 * (0.0112)	0.0290 ** (0.0130)	0.0252 ** (0.0101)	0.0266 ** (0.0112)	−0.0156 (0.0148)
Green Space Rate of Built-up Area	−0.0016 (0.0086)	−0.0123 ** (0.0052)	−0.0050 (0.0042)	−0.0108 ** (0.0048)	−0.0086 * (0.0050)	0.0030 (0.0054)	−0.0169 *** (0.0062)
Domestic Garbage Harmless Treatment Rate	−0.0001 (0.0011)	0.0002 (0.0007)	0.0011 (0.0008)	−0.0001 (0.0007)	0.0006 (0.0008)	−0.0017 * (0.0009)	0.0008 (0.0011)
Sewage Treatment Rate	−0.0013 (0.0016)	−0.0027 *** (0.0010)	−0.0036 *** (0.0010)	−0.0036 *** (0.0010)	−0.0033 *** (0.0011)	−0.0019 (0.0018)	−0.0035 *** (0.0013)
Parameter *ρ*		0.4147 *** (0.1185)	0.4310 *** (0.0658)		0.2554 *** (0.0552)	0.4845 *** (0.0631)	0.4650 *** (0.0843)
Parameter *λ*		0.2695 (0.1981)		0.7242 *** (0.0855)			
LR Test: Individual Effect	53.62 *** (0.0000)	9.86 (0.9093)	8.12 (1.0000)	24.37 * (0.0817)	9.06 (0.9111)	6.38 (0.8745)	8.11 (0.9454)
LR Test: Time Effect	420.92 *** (0.0000)	419.06 *** (0.0000)	300.79 *** (0.0000)	434.81 *** (0.0000)	408.42 *** (0.0000)	139.00 *** (0.0000)	202.68 *** (0.0000)
Hausman Test	28.64 *** (0.0074)		1.62 (0.9999)	18.47 (0.1403)	21.78 * (0.0589)	27.86 *** (0.0095)	7.39 (0.9188)
Individual Effect	Yes	Yes	—	—	—	Yes	—
Time Effect	Yes	No	—	—	—	No	—
Within *R*^2^	0.8523	0.8080	0.8671	0.7839	0.8190	0.9211	0.8390
Sample Size	217	217	217	217	217	91	126

Note: All estimation results are estimated by robust standard error method. *, **, ***, respectively, indicate that the original hypothesis is rejected at the significance level of 10%, 5%, and 1%. The samples of SAR-2 (FE) are from eastern and northeastern provinces of China. The samples of SAR-3 (RE) are from provinces in central and western China. FE and RE represent the fixed effect model and random effect model, respectively. This study has selected fixed effect or random effect according to the Hausman test results, and decided whether to control individual effect and time effect according to the LR test.

**Table 5:** LR test results (SAR/SEM/SDM).

Variable	Statistic	*p* Value
LR Test of SDM and SAR	139.90 ***	0.0000
LR Test of SDM and SEM	200.94 ***	0.0000

Note: *** indicate that the original hypothesis is rejected at the significance level of 1%.

**Table 6:** Spillover effect analysis (using the Spatial Durbin Model).

Variable Name	Direct Effect	Spillover Effect	Total Effect
Per Capita GDP	−0.0826 (0.0507)	−0.3392 (0.1161) ***	−0.4218 (0.1415) ***
Industrialization Level	0.0074 (0.0016) ***	0.0139 (0.0045) ***	0.0212 (0.0050) ***
Population Density	0.0664 (0.0224) ***	0.0670 (0.0494)	0.1335 (0.0475) ***
Percentage of Forest Cover	−0.0033 (0.0016) **	−0.0194 (0.0037) ***	−0.0227 (0.0033) ***
Total Emissions of SO_2_	0.0179 (0.0107) *	−0.0106 (0.0239)	0.0073 (0.0236)
Green Space Rate of Built-up Area	−0.0038 (0.0046)	0.0121 (0.0139)	0.0083 (0.0157)
Sewage Treatment Rate	−0.0037 (0.0011) ***	−0.0023 (0.0045)	−0.0060 (0.0052)

Note: *, **, ***, respectively, indicate that the original hypothesis is rejected at the significance level of 10%, 5%, and 1%.

## Data Availability

The Air Quality Index (AQI) data comes from the national urban air quality real-time release platform of the China National Environmental Monitoring Centre (the website is “http://www.cnemc.cn/”), and data are updated daily (final accessed on 18 May 2022). Other statistical data is from the National Bureau of Statistics (the website is “http://www.stats.gov.cn/” and final accessed on 13 June 2022) and EPS data platform (the website is “https://www.epsnet.com.cn/index.html#/Index” and final accessed on 23 August 2022).
